# A case study of Powassan virus encephalitis in an immunocompromised host

**DOI:** 10.1128/asmcr.00044-26

**Published:** 2026-07-20

**Authors:** Myriam Shehata, Gracen Betts, Sarah Chung, Anne Piantadosi, Sujit Suchindran, Daniel O. Espinoza, Matthew H. Collins

**Affiliations:** 1 Departments of Medicine and Pediatrics, Emory University School of Medicine 234195, Atlanta, Georgia, USA; 2 Emory University School of Medicine 12239, Atlanta, Georgia, USA; 3 Department of Pathology and Laboratory Medicine, Emory University School of Medicine 189273, Atlanta, Georgia, USA; 4 Department of Medicine, Division of Infectious Diseases, Emory University School of Medicine 12239, Atlanta, Georgia, USA; Rush University Medical Center, Chicago, Illinois, USA

**Keywords:** Powassan virus, encephalitis, neutralizing antibody testing, tick-borne infection, arbovirus, emerging infections, serological cross-reactivity

## Abstract

**Background:**

Powassan virus (POWV) is a tick-borne, neurotropic arbovirus endemic to Canada and the United States and is transmitted by Ixodes ticks, which also carry Lyme disease. It is an increasingly recognized cause of neuro-invasive infection in humans, for which treatment is supportive. Prognosis in POWV encephalitis is variable, ranging from complete recovery to death. As with other arboviral encephalitides, the mainstay of diagnosis is serological testing. However, molecular diagnostics play an increasingly important role, particularly in the case of immunocompromised hosts.

**Case Summary:**

A 47-year-old woman with rheumatoid arthritis taking methotrexate experienced a tick bite, followed by target-shaped rash. She was clinically diagnosed with Lyme disease and started on doxycycline therapy. She then developed headache, ataxia, dysarthria, and diplopia. An extensive infectious workup was notable for positive Powassan virus IgM confirmed with neutralizing antibody testing several weeks later. CSF Powassan virus PCR was negative. She was treated empirically for neuroborreliosis and later with pulse-dose steroids and intravenous immunoglobulin (IVIG). She ultimately recovered after a 10-day hospitalization and 5 days of inpatient rehabilitation.

**Conclusion:**

Physicians should be aware of POWV, an underrecognized cause of encephalitis, and of the complexities in diagnostic testing. Notably, cross-reactive serologies are common in arboviral infection. Serological testing remains first line in diagnosis but requires confirmatory testing typically performed at reference laboratories.

## INTRODUCTION

We present the case of a middle-aged woman taking methotrexate for rheumatoid arthritis, who developed Powassan virus (POWV) encephalitis after a tick bite. POWV is an emerging tick-borne neurotropic arbovirus endemic to the United States and Canada. Disease severity ranges from subclinical infection to fulminant encephalitis. As with other arboviruses, such as West Nile virus and St. Louis encephalitis, serological testing is first line for diagnosis; however, interpretation of serologies can be challenging due to the likelihood of cross-reactivity with other arboviruses, as well as the potential for false-negative testing in immunocompromised hosts, as in our case. Here, we outline the nuances of diagnosis of POWV encephalitis, notably the use of neutralizing antibody testing.

## CASE PRESENTATION

**Fig 1:**
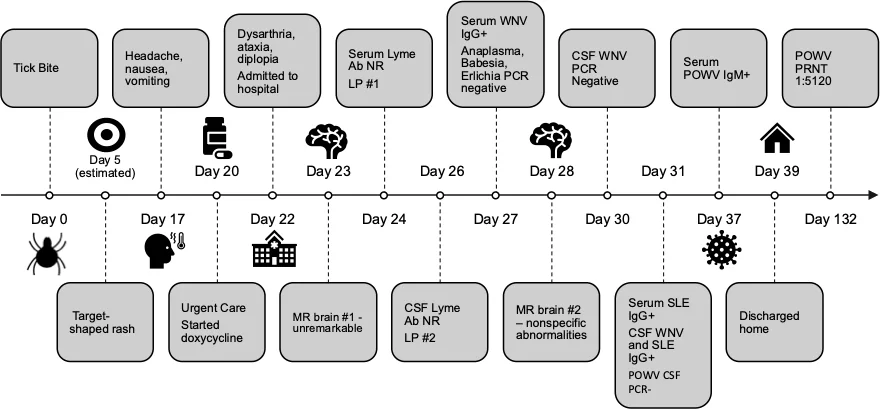
Timeline of clinical course. Events are depicted sequentially, with the time progressing left to right continuously on the horizontal line from the tick bite on Day 0; time is not to scale. Note that the time stamp on test results corresponds to the day the information became available to the clinical team. All specimens for serological testing were collected between days 22 and 33, except the POWV PRNT, which was sent on day 55. Unique abbreviations in the figure legend: NR, non-reactive.

A 47-year-old woman with rheumatoid arthritis treated with methotrexate presented to an urgent care center with a 3-day history of frontotemporal headache, nausea, and vomiting. She had experienced a tick bite to her inner thigh while on a road trip in Minnesota 3 weeks prior, followed by a target-shaped rash around the area of the bite. At the urgent care center, she was clinically diagnosed with Lyme disease and treated with oral doxycycline 100 mg twice daily. Two days later, she experienced acute onset horizontal diplopia, dysarthria, and unsteady gait, prompting presentation to our hospital (Fig. 1).

On admission, her exam was significant for marked dysarthria, wide-based ataxic gait, patellar hyperreflexia, and dysdiadochokinesia. Complete blood count with differential and comprehensive metabolic panel were within normal range. Lumbar puncture on hospital day 2 revealed an opening pressure of 23 cm H_2_O (reference range [RR] <25 cm H_2_O), with elevated total protein of 123 mg/dL (RR 15–45 mg/dL) and 113 nucleated cells per microliter (RR 0–5/uL), of which 88% were lymphocytes (RR 40–80%). Repeat lumbar puncture on hospital day 3 (performed for additional diagnostic testing) was notable for total protein of 142 mg/dL and 47 nucleated cells, with 93% lymphocytes. She had a normal continuous EEG study. ECG demonstrated an incomplete right bundle branch block, with no prior ECG for comparison and no subsequent ECG. Oral doxycycline was continued, and ceftriaxone was started due to suspicion for neuroborreliosis. On hospital day 2, her dysarthria, ataxia, and dysmetria worsened, and due to concern for worsening encephalopathy, she was transferred to the neurocritical care unit for closer monitoring. There she received IVIG (2 g/kg over 3 days) and 5 days of IV methylprednisolone. While magnetic resonance imaging (MRI) brain was unremarkable on admission, repeat MRI on hospital day 6 revealed scattered nonspecific cerebellar changes on T2/FLAIR sequences, not consistent with inflammation nor edema. In total, she completed 4 days of ceftriaxone (2 g IV every day) and 3 weeks of doxycycline therapy (100 mg orally twice per day).

**Fig 2:**
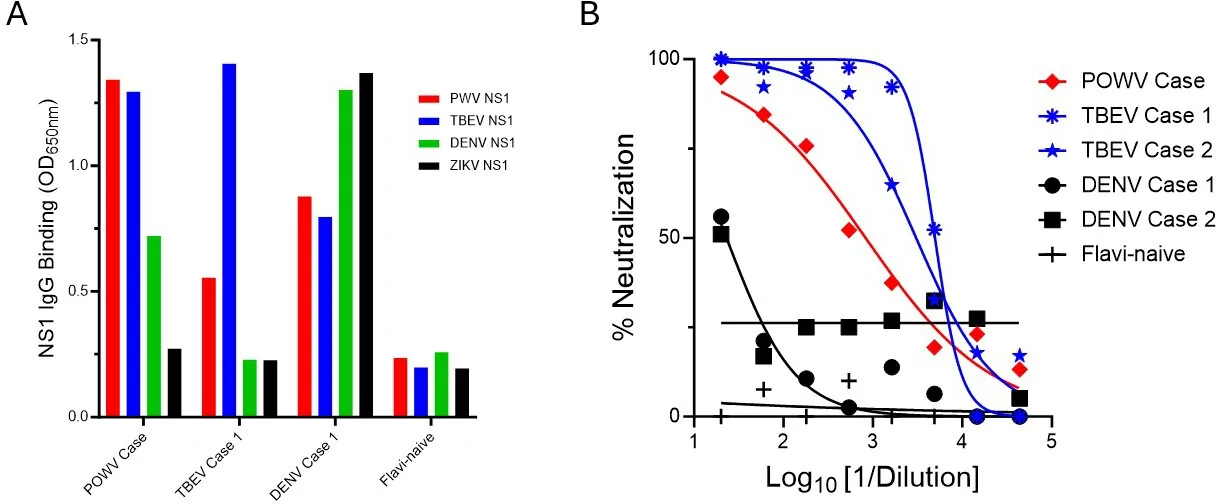
Research serological studies for POWV and related flaviviruses. (**A**) IgG binding to NS1 protein from flaviviruses indicated in the legend was assessed by an antigen-coating indirect ELISA. Serum from our patient (POWV case) and samples from additional samples representing other flavivirus exposure histories were tested as indicated on the *x*-axis. The POWV case clearly reacts most strongly to POWV NS1, with considerable cross-reactivity to NS1 from the closely related tick-borne encephalitis virus (TBEV) and less reactivity to NS1 from more distantly related flaviviruses (dengue [DENV] and Zika [ZIKV]). The other samples demonstrate a similar reactivity in accordance with exposure history and relatedness of the heterologous flavivirus antigens tested. The TBEV case serum recognized TBEV NS1 most strongly, and the DENV case serum recognizes DENV and ZIKV NS1 most strongly, as DENV and ZIKV are closely related. Reactivity of the flavivirus-naïve serum represents assay background signal as a negative control. (**B**) Neutralizing antibody titers were determined against Langat virus, which is a BSL-2 compatible flavivirus closely related to TBEV and POWV. Serum from the POWV case strongly neutralizes this virus in a focus reduction neutralization test (FRNT) with a 50% neutralization titer (FRNT50) of 827. Serum from two TBEV cases is included as a positive control and demonstrates high FRNT50: TBEV case 1 (4933) and TBEV case 2 (3036). Two DENV cases and the flavivirus-naïve negative control all exhibit FRNT50 at or below the limit of detection, consistent with the greater specificity of neutralizing antibody measures compared to binding IgG measures by ELISA: DENV case 1 (23), DENV case 2 (<20), and Flavi-naïve (<20). POWV, Powassan virus; TBEV, tick-borne encephalitis virus; DENV, dengue virus; ZIKV, Zika virus; Flavi-naive, flavivirus-naive; OD, optical density; and NS1, non-structural protein 1.

An extensive infectious workup (see Table 1) identified Powassan virus (POWV) encephalitis as the most likely diagnosis based on positive serum IgM and plaque reduction neutralization test (PRNT). Serum and CSF samples were also positive for West Nile virus (WNV) IgG and St. Louis encephalitis (SLE) IgG, but with negative corresponding tests for IgM and no PRNT performed. Further serological assays performed on convalescent serum in an arbovirology research laboratory in Atlanta, Georgia, were consistent with recent POWV infection (Table 1, Fig. 2). IgG binding to non-structural protein 1 (NS1) was measured using antigen-coating indirect ELISA. Serum from the patient reacted most strongly to POWV NS1, in addition to closely related tick-borne flaviviruses. Further neutralizing antibody testing was completed using a flavivirus closely related to POWV, Langat virus. Serum from the POWV patient strongly neutralized this virus. These results are consistent with expected results in a POWV case.

**TABLE 1:** Summary of diagnostic tests^*a*^

Negative	Positive
WNV serum and CSF IgM	WNV serum and CSF IgG
WNV CSF PCR	
SLE serum and CSF IgM	SLE serum and CSF IgG
Powassan virus (POWV) CSF PCR	POWV serum IgM; POWV serum PRNT—1:5,120 (performed at CDC); research serologies: strongly positive IgG against POWV NS1 and TBEV NS1; moderate reactivity with DENV NS1, negative reactivity to ZIKV NS1; FRNT50 = 827 against Langat virus (a BSL2 virus closely related to POWV)
California encephalitis serum and CSF IgG and IgM	
Eastern equine encephalitis serum and CSF IgG and IgM	
Western equine encephalitis serum and CSF IgG and IgM	
CSF Biofire Meningitis/Encephalitis Multiplex PCR Panel	
Serum and CSF *Borrelia burgdorferi* antibody	
Serum *Borrelia miyamoto* IgG and IgM	
Rapid malaria antigen test and thin smear	
*Babesia* species, including *B. microti*, PCR	
*Anaplasma phagocytophilum* PCR	
*Ehrlichia chaffeensis*, *ewingii/canis*, and *muris*-like PCR	
Autoimmune encephalitis panel	
Serum and CSF cryptococcal antigen	
CSF culture	
HIV 4th generation Ag/Ab	
RPR	

^*a*^ WNV, West Nile virus; SLE, Saint Louis encephalitis; CSF, cerebrospinal fluid; PCR, polymerase chain reaction; HIV, human immunodeficiency virus; RPR, rapid plasma reagin; POWV, Powassan virus; ZIKV, zika virus; NS1, non-structural protein 1, FRNT50, focus reduction neutralization titer of 50%; and BSL2, biosafety level 2.

Her symptoms started to improve 1 week after hospitalization, though with remaining dysarthria and cognitive impairment. After a 10-day hospitalization and 5 days of inpatient rehabilitation, she was discharged home. At that time, she remained with mild dysarthria but was independent in her activities of daily living. At her follow-up appointment 2 months after hospitalization, she reported being almost completely back to her baseline.

## DISCUSSION

### Powassan virus epidemiology

POWV is a tick-borne, neurotropic orthoflavivirus endemic to Canada and the United States, particularly the Northeast and Upper Midwest (1–3). It is an icosahedral, enveloped virus with a positive-sense, single-stranded RNA genome classified into two lineages, which differ in nucleotide identity by approximately 15% (1, 2, 4, 5). Both lineages are carried by *Ixodes* species ticks: lineage I is transmitted by *Ixodes cookei* and *marxi* species, neither of which frequently bite humans. Lineage II is transmitted by *Ixodes scapularis*, which carries *Borrelia burgdorferi*, the pathogenic agent in Lyme disease (1). Co-infection can occur, though it has been described rarely (1, 6). Unlike other pathogens carried by the Ixodes tick, POWV can be transmitted in as little as 15 min after a tick feeding (7).

In endemic regions of the United States, POWV has an estimated prevalence of 0–6% in *Ixodes scapularis* ticks (1, 4). POWV seropositivity has been demonstrated in multiple mammalian species: in one study, seroprevalence in white-tailed deer in Connecticut increased from 4% in 1979 to over 90% in 2009 (8). Other studies found seroprevalence in white-tailed deer ranging from 3.8 to 20%, and POWV seropositivity has been documented in foxes, wild birds, and several rodents (9). These data suggest that circulation of POWV may be much greater than previously appreciated in environments where humans may be exposed or become infected. Most reported cases in humans are neuroinvasive infections; the incidence of non-neuroinvasive or subclinical infections is unknown (1).

### Clinical manifestations and imaging findings

In the case of neuroinvasive disease, nonspecific symptoms, such as fever, malaise, and headache, appear first after an incubation period of 1–35 days (2 weeks on average) (1, 7, 9). Patients then develop neurologic signs: in one review of 73 hospitalized patients, 45% became confused; 44% had generalized weakness; 37% became encephalopathic; and 34% developed focal deficits (1).

CSF analysis often demonstrates mild-to-moderate protein elevation with lymphocytic pleocytosis and normal glucose (1, 10, 11). No peripheral laboratory abnormalities are classically associated with POWV infection, though thrombocytopenia has been reported (1, 10, 11). As with other arboviral infections, treatment is supportive, though immunosuppression and immunomodulation may be considered in the absence of contraindication. Some case reports have demonstrated positive outcomes with steroids and IVIG, though this has not been studied rigorously (1, 6).

Prognosis is variable, from complete recovery to fulminant encephalitis and death in 10% of cases (1, 3). Half of survivors experience persistent neurologic deficits after recovery, including speech and cognitive difficulties, imbalance, and ophthalmoplegia (7, 9).

There are no characteristic imaging findings in POWV encephalitis. Case reports have documented a range of findings on brain MRI, from unremarkable to diffusion restriction due to cytotoxic edema to T2/FLAIR hyperintensity in the cerebellum and posterior fossa (3, 12). Abnormalities are most common in the cerebellum and deep gray structures (7). It has been hypothesized that cerebellar or posterior fossa lesions may represent a poor prognostic factor, portending risk of brain herniation (1, 12, 13). In one review, cerebellar findings in POWV encephalitis were identified in “70% of fatal cases compared to less than 20% of non-fatal cases (*P* = 0.0004)” (1). Thus, while the interpretation of imaging findings is not straightforward in suspected arboviral encephalitides, brain MRI provides useful data and may aid in prognostication.

### Interpretation of diagnostics

Clinicians suspecting an arboviral infection can access useful guidance and resources via their state public health lab and the Arboviral Diseases Branch of the Centers for Disease Control and Prevention (CDC), which provides clinical and diagnostic support for suspected arboviral infections (14). The CDC diagnostic criteria for an arboviral infection require a corresponding clinical syndrome plus:

1. Direct detection of virus by culture, antigen, or nucleic acid amplification test, or
2. A fourfold rise in antibody titers between acute- and convalescent-phase sera, or
3. Virus-specific IgM detection in CSF, with confirmation by neutralizing antibody test or negative IgM to other endemic arboviruses, or
4. Virus-specific IgM detection in serum, with confirmation by neutralizing antibody test (10, 15).

In an immunocompromised host, it is reasonable to include molecular tests (RT-PCR or metagenomic sequencing) for direct pathogen identification due to the possibility of false-negative serological testing (7). This should be considered especially in patients on B-cell targeting immunotherapy or with impaired B-cell response (7). It is also reasonable to attempt molecular diagnostics in any patient in the first week after symptom onset, during which antibody response may be unreliable (7).

In our patient, PCR testing was negative. For POWV and other arboviral encephalitides, viral replication is thought to be generally short-lived, and neuro-invasive symptoms often occur *after* replication has waned. Therefore, PCR testing may not be useful unless specimens are gathered early in the disease course. The window for POWV RNA detection in humans has not been precisely determined (3, 7, 10, 12). Data extrapolated from other arboviruses, particularly WNV, suggest urine may be more sensitive for viral detection than CSF or serum, though this has not been demonstrated in the case of POWV (7, 10). Metagenomic next-generation sequencing of CSF samples can be used to diagnose POWV encephalitis; however, this method is subject to the same limitations as PCR testing and remains a relatively costly method; therefore, it is typically reserved for cases in which other tools have not yielded a diagnosis (7, 10). Serological testing remains first-line for the diagnosis of arboviral encephalitides.

Virus-specific IgM with a corresponding clinical syndrome suggests a *probable* diagnosis, which can be confirmed by the detection of neutralizing antibody in CSF or serum or a fourfold rise in titers in convalescent serum (7, 10, 15). For neuroinvasive disease, diagnosis can also be made by positive CSF IgM for the virus in question if accompanied by *negative* IgM serologies to other arboviruses endemic to the region where exposure occurred (10, 15). Expert opinion suggests that when pre-test probability is high, if initial serologies are negative, testing should be repeated between illness days 5 and 15; seroconversion at that time is confirmatory (10).

A complicating factor in the diagnosis of arboviral infection is the potential for antibody cross-reactivity between related viruses. Our patient had positive WNV and SLEV IgG in CSF and serum. In the absence of detectable IgM to either of these pathogens, these findings were favored to represent cross-reactivity, though prior infection is also possible (10, 15). Though, classically, IgM is understood to indicate acute or recent infection, arboviral IgM can persist for months to years after infection; in these cases, quantitative titer evaluation may be needed to determine the causative virus (15). In our case, this was less of a concern given the sole positive IgM result (POWV) and strongly positive confirmatory PRNT test.

### Neutralizing antibody testing

Neutralizing antibody testing is the gold standard confirmatory test for arboviral infection and highly specific in diagnosis (7, 10). The classic and most widely used neutralizing antibody technique is plaque reduction neutralization testing (PRNT). PRNT provides a quantitative measure of the ability of antibodies present in a sample (serum or CSF) to inhibit cellular infection when virus is added to a growing cell line in culture (7, 10). This test is typically only performed at public health reference laboratories, requiring level 3 biosafety precautions for some viruses like POWV (10, 15, 16). Though the most specific test for arboviral infection, it is not the most sensitive, as IgM is not always neutralizing early in the acute phase of an immune response; therefore, neutralization testing is ideally performed on a convalescent sample and typically used as a confirmatory test after a more sensitive assay is suggestive of infection (7). While it cannot assess the timing of infection (neutralizing antibodies persist in serum for years to decades), PRNT is particularly useful in cases where antibody cross-reactivity (e.g., between flaviviruses) impedes accurate diagnosis (10, 16).

### Conclusion

POWV encephalitis is an underrecognized cause of encephalitis and should be considered early in patients who present with progressive neurologic symptoms, particularly those with a history of tick bite and/or time spent in endemic regions. Molecular diagnostics (most commonly RT-PCR) should be considered in diagnosis, particularly for immunocompromised patients or patients who present early in their disease course, in addition to serological testing. Negative molecular diagnostics do not exclude the diagnosis of POWV encephalitis. Positive serological testing typically requires confirmatory testing, either by a four-fold rise in antibody titers or neutralizing antibody testing. Confirmatory testing can help differentiate true positive serological tests from cross-reactivity, which is common among arboviruses. Brain MRI should be considered as part of the diagnostic workup, and while there are no characteristic features of POWV, cerebellar findings are most common and may represent a poor prognostic factor.

Treatment for POWV encephalitis is supportive, though immunosuppression and immunomodulation can be considered in the absence of contraindication.
